# Effects of Chronologic Age and Young Child Exposure on Respiratory Syncytial Virus Disease among US Preterm Infants Born at 32 to 35 Weeks Gestation

**DOI:** 10.1371/journal.pone.0166226

**Published:** 2016-11-29

**Authors:** Eric A. F. Simões, Evan J. Anderson, Xionghua Wu, Christopher S. Ambrose

**Affiliations:** 1 University of Colorado School of Medicine, Colorado School of Public Health and Children’s Hospital Colorado, Aurora, CO, United States of America; 2 Departments of Pediatrics and Medicine, Emory University School of Medicine, Atlanta, GA, United States of America; 3 Department of Biostatistics, MedImmune, Gaithersburg, MD, United States of America; 4 AstraZeneca, Gaithersburg, MD, United States of America; Kliniken der Stadt Köln gGmbH, GERMANY

## Abstract

**Objective:**

To estimate the incidence of respiratory syncytial virus (RSV) disease as a function of chronologic age and exposure to young children in US preterm infants.

**Methods:**

In the RSV Respiratory Events among Preterm Infants Outcomes and Risk Tracking (REPORT) study, preterm infants born at 32–35 weeks gestational age (wGA) were enrolled from 188 US clinics and followed September-May of 2009–2010 or 2010–2011. Infants with medically-attended acute respiratory illness had nasal/pharyngeal swabs collected for viral testing. Results of RSV tests conducted during routine clinical care were also collected. Event rates during November-March were modeled as a function of chronologic age and birth month using Poisson regression and adjusting for other covariates. Rates were calculated overall and for infants with and without exposure to young siblings or daycare attendance. Of 3317 infants screened, 1646 were enrolled as a consecutive sample. Infants with chronic lung disease of prematurity, hemodynamically significant congenital heart disease, life expectancy <6 months, or receiving or being considered for RSV immunoprophylaxis were excluded. 84% of patients completed the study. Demographics of the enrolled cohort were generally similar to those of US infants born at 32–35 wGA; infants 32–34 wGA, Hispanic infants, and infants of less-educated mothers were under-represented.

**Results:**

Among 1642 evaluable infants, outpatient RSV lower respiratory illness incidence was highest at older ages, whereas RSV hospitalization and intensive care unit (ICU) admission were highest at younger ages. In all instances, young child exposure was associated with higher RSV incidence. The highest RSV hospitalization and ICU rates occurred among February-born infants with young child exposure, at 19.0 (95% CI, 13.5–27.0) and 6.5 (95% CI, 5.6–7.6) per 100 infant-seasons, respectively.

**Conclusions:**

Preterm infants have a substantially elevated risk of RSV disease. Young age and exposure to other young children identify those at greatest risk of severe RSV disease.

**Trial Registration:**

Clinicaltrials.gov: NCT00983606.

## Introduction

Respiratory syncytial virus (RSV) is the most important viral pathogen causing acute lower respiratory illness (LRI) and community-acquired pneumonia in young children [1,2]. Several medical conditions increase the risk of severe RSV LRI in young children, including chronic lung disease of prematurity (CLDP), hemodynamically significant congenital heart disease (CHD), and preterm birth at ≤35 weeks gestational age (wGA). Young chronologic age has been recognized as a major risk factor for severe RSV disease, with more than 80% of RSV hospitalizations occurring in infants younger than 12 months and the highest rates observed in the first 5 months of life [3–7]. The risk of laboratory-confirmed RSV hospitalization among US infants was estimated in a large, multi-season active surveillance study, with the highest rate of 25.9 per 1000 infants per season observed in the second month of life [5]. Additionally, significant exposure to young children through living with young siblings or daycare attendance has also been consistently associated with an increased RSV risk among preterm infants [6,8–12].

The prospective RSV Respiratory Events Among Preterm Infants Outcomes and Risk Tracking (REPORT) study was designed to determine the incidence of medically-attended, laboratory-confirmed RSV disease among US preterm infants born at 32 to 35 wGA, and to identify risk factors associated with RSV disease within this population (NCT00983606) [13]. Subsequent to the original publication of the REPORT results, the American Academy of Pediatrics Committee on Infectious Diseases updated their guidance on the use of RSV immunoprophylaxis, recommending against use in infants born at 29 to 35 wGA without underlying comorbidity. This was based in part on the assertion that available data did not indicate a significant difference in hospitalization rates for RSV infection between preterm infants born at 29 to 36 wGA without underlying comorbidity and full-term infants [14,15]. Given the demonstration that hospitalization incidence will vary substantially based on infant age and change as infants age through the RSV season [5,16], we analyzed data from the REPORT study to evaluate the risk of RSV-related outpatient LRI, hospitalization, and intensive care unit (ICU) admission as a function of chronologic age among US preterm infants with and without significant exposure to young children, based on birth month and age of exposure.

## Materials and Methods

Preterm infants born between 32 weeks, 0 days and 35 weeks, 6 days GA and ≤6 months of age (birth in May through February) were enrolled from 188 US clinics in 38 states and followed from September to May of 2009–2010 or 2010–2011 [13]. Infants with CLDP, hemodynamically significant CHD, life expectancy <6 months, or receiving or being considered for RSV immunoprophylaxis were excluded. Infants who presented with medically-attended acute respiratory illness in the outpatient setting had nasal and pharyngeal swabs collected according to the study protocol and were tested for RSV, adenovirus, parainfluenza virus (PIV) types 1–3, human metapneumovirus (hMPV), and influenza A and B using quantitative reverse transcriptase polymerase chain reaction (Prodesse® ProFlu™+, Hologic Gen-Probe Incorporated, San Diego, CA). Results of RSV tests conducted as a part of routine clinical care, including during hospitalizations, were also collected. For respiratory-related emergency department visits and hospitalizations without RSV testing, the event was considered RSV-related if an RSV-positive sample collected by study personnel within seven days of the emergency department visit or hospitalization identified RSV. Written informed consent and any locally required authorization were obtained from the child's parent or guardian before performing any protocol-related procedures.

Event rates for outpatient LRI due to all viruses tested, RSV-related hospitalizations, and RSV-related ICU admissions were calculated for infants with and without significant young child exposure, defined as daycare attendance or living with non-multiple birth preschool-age siblings. Event rates were calculated per 100 infant-seasons, with a season defined as 5 months of observation from November 1 to March 31. Data were analyzed by chronologic age and birth month, as both have been used as markers of risk in previous studies and in RSV immunoprophylaxis policy [8–12,14].

Sample permitting, age trends were analyzed by fitting the data into a Poisson model including age at time of event or birth month and existence of young child exposure in the model, and adjusting for other significant risk factors for the specific virus and endpoint, similar to the approach described by Winterstein et al [16]. In the analysis model for RSV ICU admissions, only age at time of event or birth month and existence of young child exposure were included in the model, as there were no other significant risk factors for RSV ICU admissions [13]. The predicted values of incidence and 95% confidence interval (CI) for each age group were obtained by entering the age and observed values of other covariates into the fitted model. The average of the predicted values and 95% CI for the specific group was then calculated.

## Results

A total of 1642 preterm infants were evaluable. The mean chronologic age at enrollment was 2.3 months and 8.6%, 13%, 25%, and 54% of patients were 32, 33, 34, or 35 wGA, respectively. The chronologic and gestational age of enrolled infants were similar across enrollment months. Overall, the demographics of the enrolled study cohort were similar to those of the US cohort of infants born at 32 to 35 wGA. The primary differences were that the study cohort under-represented infants 32 to 34 wGA, Hispanic infants, and infants of less-educated mothers [13].

**Fig 1:**
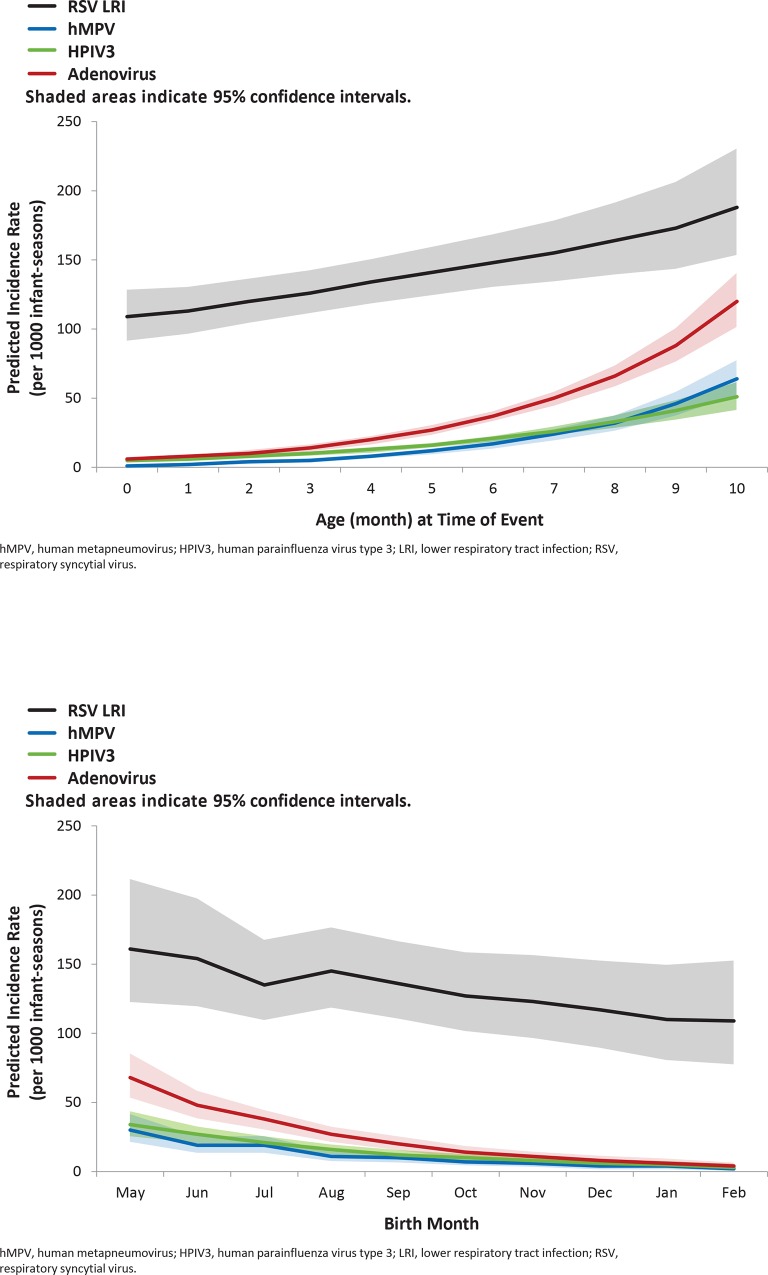
Modeled Viral Outpatient Lower Respiratory Tract Infection (LRI) Rate During Respiratory Syncytial Virus (RSV) Peak Season by A) Exposure Age and B) Birth Month.

**Fig 2:**
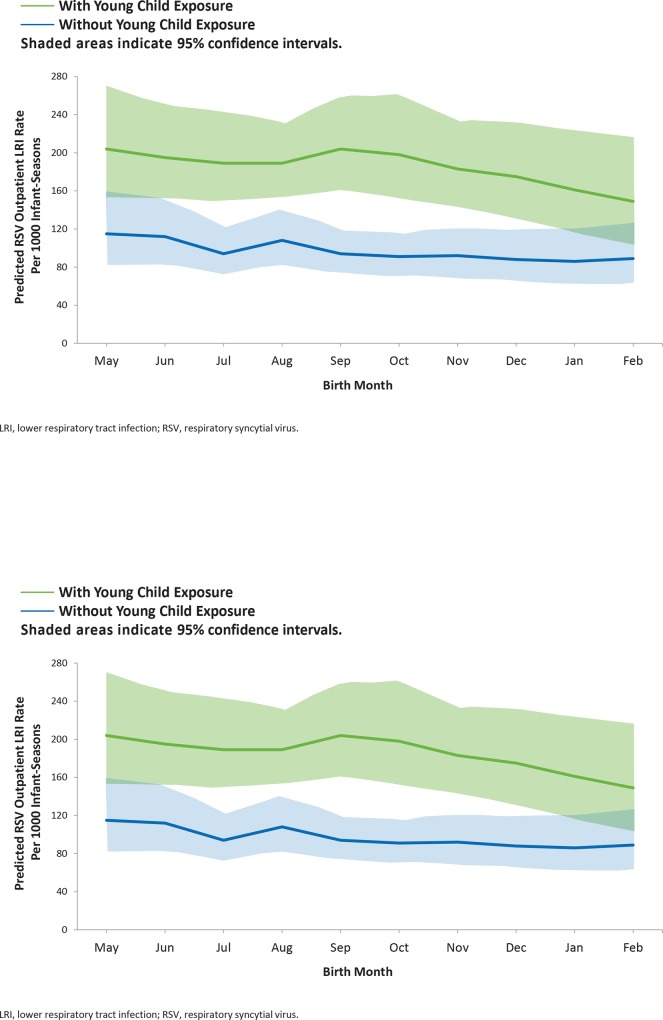
Modeled Respiratory Syncytial Virus (RSV) Outpatient Lower Respiratory Tract Infection (LRI) Rate for Infants With and Without Young Child Exposure by A) Exposure Age and B) Birth Month.

Among outpatient LRI cases, RSV predominated. The overall rates of outpatient LRI per 100 infant-seasons were 13.7 for RSV, 2.9 for adenovirus, 1.7 for PIV3, 1.3 for hMPV, 0.6 for influenza A, 0.5 for influenza B, 0.3 for PIV1, and 0 for PIV2. The number of cases of RSV, adenovirus, PIV3, and hMPV were sufficient to enable modeling. For all viruses modeled, outpatient LRI incidence increased with increasing age (**Fig 1A and 1B**). Overall, young child exposure was associated with a 3.3-fold higher risk of adenovirus and a 2.0-fold higher risk of RSV and hMPV; no association was observed for PIV3. Outpatient RSV-related LRI incidence was highest in infants with young child exposure who were 10 months of age or born in May, with rates of 22.7 (95% CI, 18.6–27.7) and 20.4 (95% CI, 15.4–27) per 100 infant-seasons respectively (**Fig 2A and 2B**).

**Fig 3:**
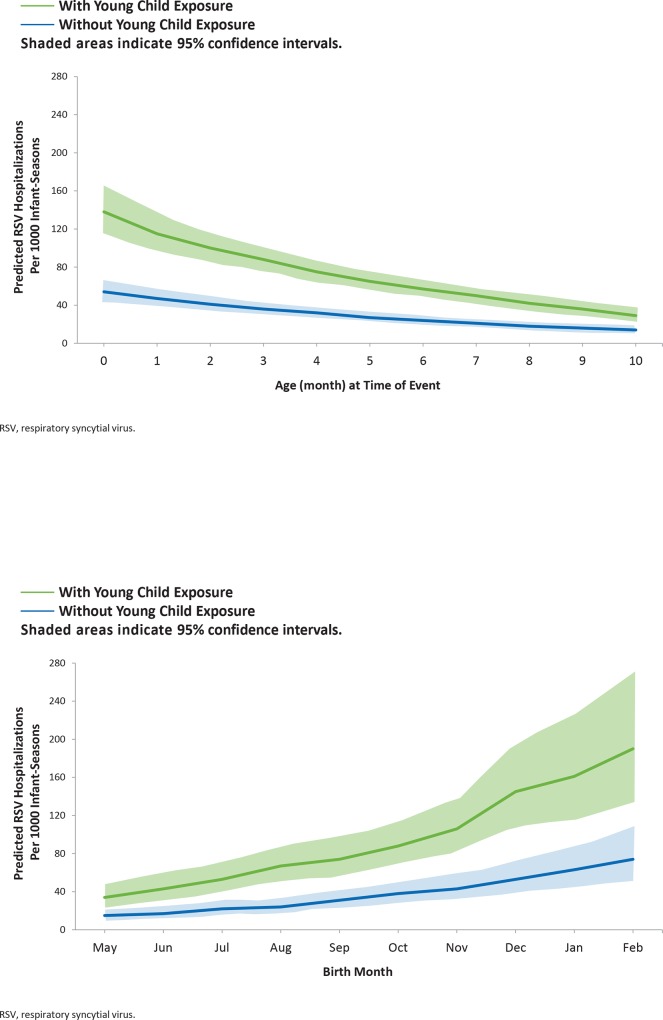
Modeled Respiratory Syncytial Virus (RSV) Hospitalization Rate for Infants With and Without Young Child Exposure by A) Exposure Age and B) Birth Month.

In contrast to outpatient LRI incidence, RSV hospitalization incidence was highest among the youngest infants. Among all infants, the incidence of RSV hospitalization ranged from 8.2 (95% CI, 6.9–9.7) to 2.3 (95% CI, 1.8–2.9) per 100 infant-seasons among those <1 and 10 months of age, respectively. By birth month, the range of rates was similar, increasing from 2.5 (95% CI, 1.7–3.5) to 10.8 (95% CI, 7.7–15.3) per 100 infant-seasons among those born in May before the RSV season and February during the RSV season, respectively. As with outpatient LRI, RSV hospitalization incidence was higher at all ages (**Fig 3A**) and birth months (**Fig 3B**) among infants with young child exposure compared to those without significant young child exposure. The highest RSV hospitalization rates were 13.8 (95% CI, 11.5–16.6) and 19.0 (95% CI, 13.5–27.0) per 100 infant-seasons, respectively, among <1-month-old infants and those born in February with young child exposure.

**Fig 4:**
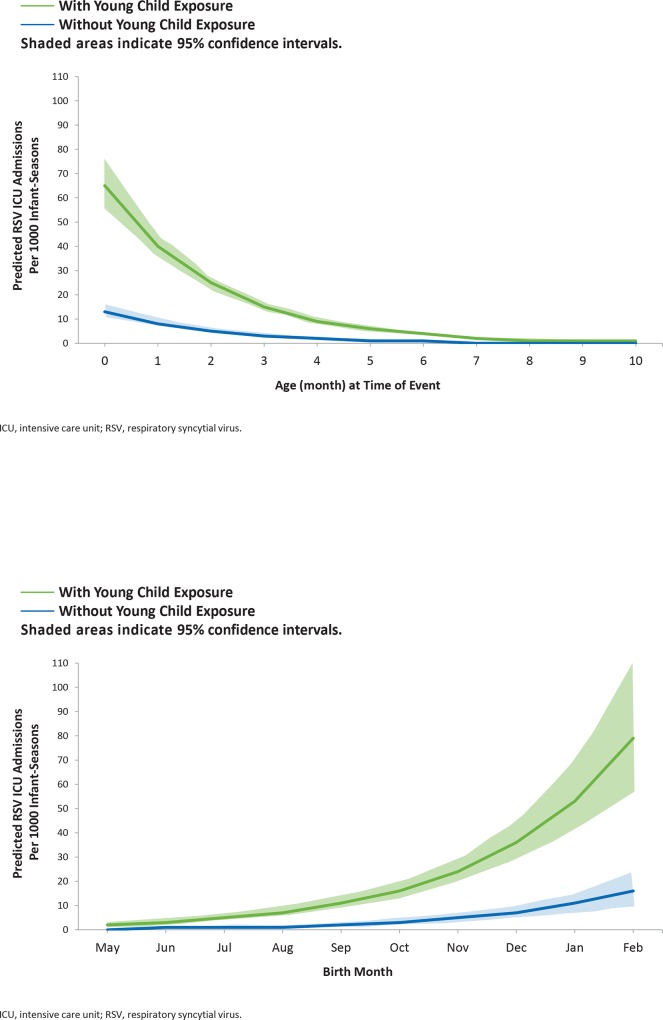
Modeled, respiratory syncytial virus (RSV) intensive care unit (ICU) Rate for Infants With and Without Young Child Exposure by A) Exposure Age and B) Birth Month.

As with RSV hospitalization, RSV ICU admission incidence was highest among the youngest infants, with an even stronger effect of young age. Among all infants, the incidence of RSV ICU admission ranged from 3.1 (95% CI, 2.7–3.6) to 0 per 100 infant-seasons among those <1 and 10 months, respectively. By birth month, the ICU admission rates increased from 0.1 (95% CI, 0.1–0.2) to 3.6 (95% CI, 2.6–4.9) per 100 infant-seasons among those born in May before the RSV season and February during the RSV season, respectively. As with other outcomes, RSV ICU admission was higher at all ages (**Fig 4A**) and birth months (**Fig 4B**) among infants with young child exposure compared to those without significant young child exposure. The highest RSV ICU admission rates were 6.5 (95% CI, 5.6–7.6) and 7.9 (95% CI, 5.7–11.0) per 100 infant-seasons, respectively, among <1-month-old infants and those born in February with young child exposure.

## Discussion

The current analysis provides contemporary US-specific estimates of laboratory-confirmed RSV disease incidence among a large, representative cohort of US preterm infants born at 32 to 35 wGA that was prospectively followed with active surveillance for RSV disease. The results confirm that RSV is the predominant virus responsible for severe lower respiratory disease in these preterm infants, and, consistent with previous observations [8–10,12], young chronologic age and exposure to young children significantly increased the risk of severe RSV disease in preterm infants born at 32 to 35 wGA. The current results are unique in that they provide continuous age-based risk models for outpatient and inpatient disease for infants with and without young child exposure. In addition, the absolute risk estimates are more accurate than those derived from administrative databases with passive case ascertainment [16,17].

Outpatient LRI increased with chronologic age and was consistently higher in those with significant young child exposure, consistent with LRI risk being predominantly driven by RSV exposure. However, for RSV hospitalization and ICU admission, exposure to young children was associated with an increased risk, but the major predictor of risk was young chronologic age, consistent with severe RSV disease being driven by anatomical and physiologic vulnerability, specifically smaller airways and more immature immunologic responses in younger infants [18].

Supporting our observations regarding the effects of age on disease incidence, two other recent US studies have demonstrated associations between preterm birth, young chronologic age, and increased risk of RSV hospitalization. In 2013, Winterstein et al demonstrated that moderate preterm status (32 to 34 wGA) doubled the risk for RSV hospitalization compared to term infants of the same age, and that the risk was highest in the youngest infants [16]. Helfrich et al also identified late preterm birth, defined as 33 weeks to 36 weeks and 6 days, as an independent risk factor for RSV hospitalization, with the greatest risk observed in the first months of life as evidenced by a median age at admission of 3.2 months [17].

Some experts have stated that the risk of RSV hospitalization in infants 29 to 35 wGA is similar to that of term infants [14]. However, the current observed rates refute this assertion. The risk of laboratory-confirmed RSV hospitalization among US infants of all gestational ages during the first three months of life was estimated at 17.9 (95% CI, 15.7–20.1) per 1000 infants per season, with the highest rate of 25.9 (95% CI, 21.3–30.8) per 1000 observed in the second month of life [5]. The present study identified considerably higher rates of RSV hospitalization in 32 to 35 wGA infants, at 82 (95% CI, 69–97) per 1000 infant-seasons among those <1 month of age. The risk was further elevated among the youngest infants with exposure to young children, at 138 (95% CI, 115–166) per 1000 infant-seasons for those <1 month and 190 (95% CI, 135–270) per 1000 infant-seasons for those with young child exposure born in February. Among these youngest infants with exposure to other young children, the observed rates of RSV ICU admission were 65 (95% CI, 56–76) and 79 (95 CI, 57–110) per 1000 infant-seasons, respectively.

Strengths of this analysis include the large, prospectively recruited patient cohort that was generally similar to the US cohort of infants born at 32 to 35 wGA, inclusion of data from two RSV seasons from 188 clinical sites across the US, and active surveillance and laboratory confirmation of RSV infection in symptomatic infants. The principal limitations of this study may have led to an underestimation of the true incidence in all 32 to 35 wGA infants in the absence of RSV immunoprophylaxis. Due to the necessary exclusion of infants receiving RSV immunoprophylaxis during the 2009–2011 RSV seasons, 32 to 34 wGA infants <3 months at RSV season start, with young siblings or daycare attendance, were under-represented in the study cohort. Additionally, not all hospitalized infants were tested for RSV and other viruses, which could further underestimate RSV hospitalization and associated ICU rates, and prevented an analysis of the incidence of hospitalization and ICU admission for other viruses. An additional limitation related to the reverse transcriptase polymerase chain reaction testing of outpatient LRI cases, which did not test for rhinovirus because it was not included in the chosen assay. Finally, although it has been well established that RSV and hMPV are primary drivers of disease when detected, the causal role of adenovirus is more uncertain. Adenovirus has been identified among many healthy controls in a recent study in Africa, although not in a recent study of community-acquired pneumonia [19,20].

In conclusion, RSV disease rates were consistently higher among preterm infants with exposure to young children. As a function of age, the rate of outpatient RSV LRI increased in older preterm infants, while RSV hospitalization and ICU admission rates were highest among younger preterm infants. The observed rates of RSV hospitalization were substantially higher than those observed in recent similar studies of the general US infant population. These data demonstrate that higher risk for 32 to 35 wGA infants can be easily identified by age or birth month and significant exposure to other young children. These infants would benefit from targeted efforts to prevent severe RSV disease.

## Supporting Information

S1 File REPORT by age Data. (CSV)
