# Correction: Effects of Chronologic Age and Young Child Exposure on Respiratory Syncytial Virus Disease among US Preterm Infants Born at 32 to 35 Weeks Gestation

**DOI:** 10.1371/journal.pone.0168882

**Published:** 2016-12-13

**Authors:** Eric A. F. Simões, Evan J. Anderson, Xionghua Wu, Christopher S. Ambrose

There is an error in Fig 2. The image for Fig 2A is a duplicate of Fig 2B. The figure legend is correct. Please view the corrected Fig 2 here.

**Fig 2:**
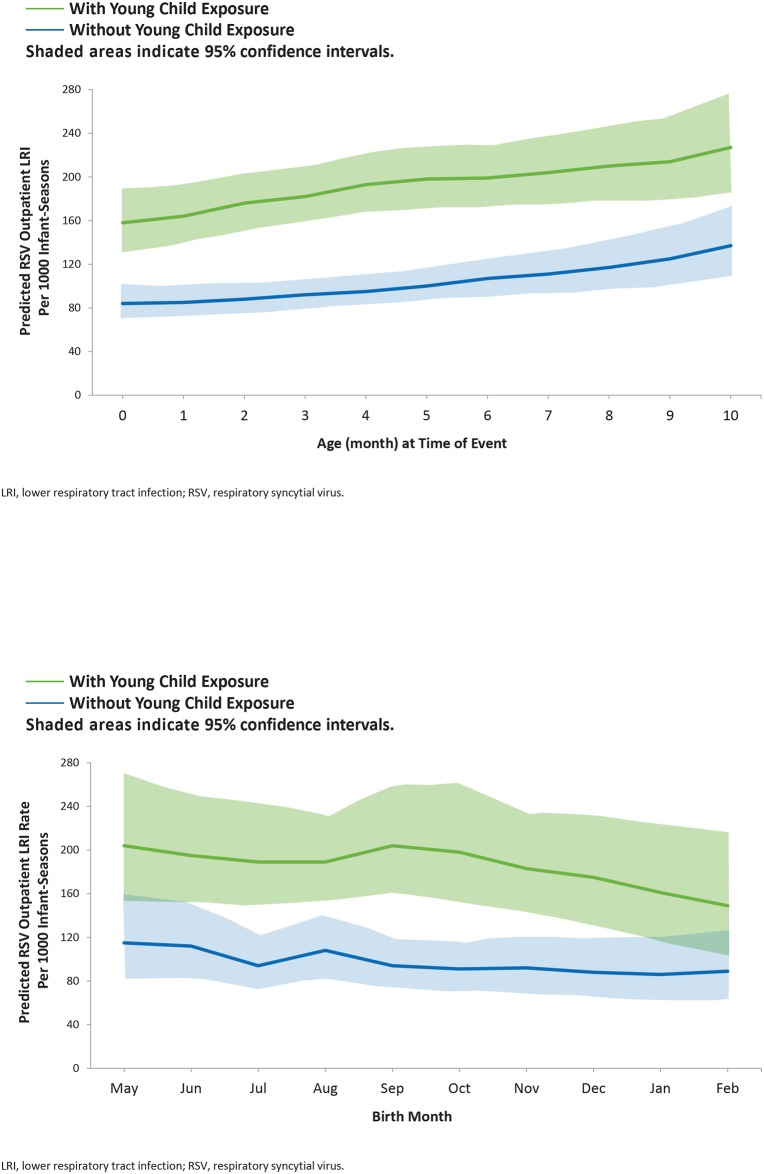
Modeled Respiratory Syncytial Virus (RSV) Outpatient Lower Respiratory Tract Infection (LRI) Rate for Infants With and Without Young Child Exposure by A) Exposure Age and B) Birth Month.
